# Surgical Club of the South West, May and October 1990 Meetings

**Published:** 1990-12

**Authors:** 


### Surgical Club of the South West

### Meeting held at Yeovil May 11th 1990 under the Chairmanship of Mr Colin Davidson in Gloucester, October 1990

### RURAL SURGERY REVISISTED

**D. A. Griffiths**
Yeovil District Hospital

The changes in one General Surgeon's practice from 1977 to 1990 were audited and analysed. The results confirmed that in the South West Region the smaller hospital general surgeon received the larger share of referrals from family practitioners. Over one quarter of GP referrals were to the general surgeons. Signficant quadrupling in the number of breast cases, gastrointestinal bleeding problems, colorectal cancers and urological problems were confirmed during this period.

These figures were due to an increasing elderly native population and immigration from outside the district. No consultant expansion or extra resources had taken place durng this period and waiting lists had increased despite increased throughput.

### THORACIC OUTLET SYNDROME

**G. S. Payne**
Yeovil District Hospital

The emphasis of the presentation was on the vascular complications of the condition, which may lead to disabling ischaemia or even gangrene of the arm.

The symptoms are produced by compression of the subclavian artery in the root of the neck as it passes from the thorax to the arm. The most common cause is cervical rib, but other causes are costoclavicular nipping, scalenus muscle bands, anomalies and fractures of the first rib or clavicle, and, further laterally, the coracoid process and pectoralis minor tendon. Rare causes such as tumours were mentioned.

Cervical rib occurs in 0.5-1pc of the population but only 10pc give rise to symptoms. Clinical assessment relies on examination and radiology. Clinical tests for the condition are notoriously unreliable with a high percentage of both false positives and negatives. Vascular problems can be identified by reduced blood pressure, a bruit or palpable aneurysm in the neck (post-stenotic dilatation). Arteriography will demonstrate the vascular conformation.

Surgical approaches to the subclavian artery are supraclavicular and trans-axillary. The advantages of each were mentioned.

### BEETHOVEN'S MEDICAL HISTORY A RHEUMATOLOGICAL REAPPRAISAL VIEWPOINT

**T. G. Dalferman**
Yeovil District Hospital

Born in Bonn in December 1770, Beethoven moved to Vienna in 1792 and died there aged 56 in February 1827. His father was an alcoholic, his adored mother died aged 40 from tuberculosis as did a younger brother. Ludwig contracted smallpox in childhood and in his teens suffered the bouts of abdominal pain which were to plague him recurrently throughout life and which at times were of a severity to prostrate him. His deafness, first recorded in 1797, progressed to total hearing loss over a quarter of a century. Bouts of depression, largely resulting from the threat his hearing loss presented to his musicianship, are well documented and suicide was possibly contemplated in 1802 as suggested by the Heiligenstadt Testament, written then but discovered posthumously.

After the decade to 1812, when his musical output had been prodigious, his health inexorably declined. His gut symptoms became more troublesome; chest symptoms, 'thoracic gout', and infections were frequent. In addition, from 1816 there are many references to attacks of rheumatism, sometimes confining him to bed. In 1823 these were associated with painful eyes and violent diarrhoea. In 1826 again rheumatism and gout struck and Beethoven mentions severe back pain which had been present for some time.

At the age of 50 jaundice first occurred. In his last four months he suffered dropsy with peripheral oedema and massive amounts of ascites, drained at paracentesis. At autopsy were recorded a shrunken liver, splenomegaly and renal calculi. His eighth nerves were atrophied, the associated arteries dilated.

A differential diagnosis includes chronic active or cryptogenic cirrhosis with resultant portal hypertension and multi-system associations. A seronegative arthropathy is likely, either post-dysenteric reactive in type or associated with inflammatory bowel disease. The development of sacro-iliitis is suggested. All this probably superimposed on irritable bowel syndrome. Paget's disease has been suggested as a cause for the deafness but the evidence suggests it was merely incidental. Finally, I hypothesise that Beethoven had sarcoidosis and propose that all his multi-system problems, including the deafness, can be explained by this diagnosis.

### PROXIMAL GASTRIC VAGOTOMY

**R. J. Clarke**
Yeovil District Hospital

Proximal gastric vagotomy (PGV) is approximately 21 years old and the safety and relative freedom from side effects, in experienced hands, compared with other operations for peptic ulcer is fairly well established. What is still more problematical is the peptic ulcer recurrence rate as recurrence can occur many years after apparently successful surgery.

Since 1970 PGV has been my standard operation for duodenal ulcer. I have therefore analysed all my PGVs (114 patients) since starting in Yeovil in 1973. All patients were prospectively followed up annually or to the death, but only 112 patients were available for assessment. Particular attention was paid to the 62 patients followed up for more than 10 years.

Overall 82% were graded Visick I or II and the reasons for unsatisfactory results were discussed. All unsatisfactory results (Visick III & IV) were endoscoped at least once. 6 patients developed DU recurrence or GU between 6 and 15 years after PGV and most recurrences were of late onset. 8% (2.5% male, 24% female) of patients followed up for 10 to 16 years had recurrence, but this tended to be less "aggressive" than the primary ulcer and could be successfully managed by $H^2$ antagonists or further surgery with a good symptomatic result.

These results are roughly in agreement with D. Johnson and others and certainly suggest that PGV in experienced hands is a good operation for DU.

### SURGEON IN NEPAL

**M. A. R. Eslick, Colonel RAMC**

This paper is based on the clinical material I saw, photographed and recorded during a period of eighteen months (1974–76) spent in the British Military Hospital in Dharan Cantonment which is in Eastern Nepal and lies in the foothills of the Himalayas housing the H.Q. of the Brigade of Gurkhas who recruit into the British Army. The hospital is modern and has 78 beds of which 32 beds are surgical enabling about 2000 operations to be performed annually, mainly on local Nepalese civilians free of charge. Roads to the South, East and West make transportation of patients easy, but precipitous mountain tracks to the North are negiotiated for up to 20 days by patients or by their relatives carrying them in hammocks or slung from poles.

Here is a summary of the major material seen:-

- Old unreduced fractures and dislocations
- Untreated osteomyelitis in all stages
- Fresh Supracondylar fractures (falls out of trees & off bullocks)
- Nasopharyngeal and GIT cancers
- Pulmonary, bone and joint Tuberculosis
- Burnt-out cases of Leprosy with severe cosmetic defects
- Buerger's disease
- Burns-mostly old contractures with severe disabilities
- Congenital Deformities eg.
  - Duplication of bowel, Sacrococcygela teratoma, Supernumary fingers and toes, Hare lips and Cleft palates
- Amoebic abscesses

Tuberculosis is so common that it always has to be excluded first in assessing patients. Spinal tuberculosis is a common crippling disease as both men and women can only find employment as porters and a healthy spine is essential. I therefore performed a lot of one stage operations of debridement and anterior spinal fusions under antibiotic cover on afflicted children with good results. Slides of a large series of cleft palate and lip operations were demonstrated. Elephant injuries, bear maulings and snake bites were also dealt with in this most remarkable and interesting setting.

### PRIMARY TUMOURS OF THE DUODENUM

**N. C. G. Bathurst, J. Virjee**
Yeovil District Hospital

Primary tumours of the duodenum are rare but have a poor prognosis when malignant because of late diagnosis. They tend to present with non-specific symptoms and need to be detectable by investigations directed at discovering the more common forms of upper gastro-intestinal pathology. A series of 11 benign and 8 malignant tumours of the duodenum, excluding involvement with lymphoma, are presented all of which were identified and correctly categorized as benign or malignant by a routine hypotonic double contrast duodenogram as part of a Barium Meal examination. This is compared with their detection and categorization by endoscopy.

Five of nine single adenomatous polyps in the duodenal cap were seen at endoscopy. The other benign tumours (a somatostatinoma and a galglioneuroma, both of the second part) were seen but biopsy failed to give diagnostic material. Endoscopy was performed in six of the eight patients with malignant tumours and repeated in one. Five of the tumours were in the second part and three in the third and fourth parts. Two examinations failed to reveal the tumour and of the five successful examiantions, diagnostic biopsies were obtained in two.

This series illustrates that the reputed advantage of endoscopy over barium studies in obtaining biopsy material is not always borne out in practice and that duodenal lesions, particularly of the third and fourth part, may be beyond the reach of the endoscope. A standard hypotonic double contrast barium study should, however, examine the whole duodenum and can be relied upon to identify and characterize benign and malignant tumours of the duodenum.

### BILIARY LITHOTRIPSY—THE SOUTHMEAD EXPERIENCE

**A. P. Barlow, J. M. Haworth, M. H. Thompson**
Southmead Hospital, Bristol

Following the first report of the successful treatment of patients with cholelithiasis by extracorporeal shockwave lithotripsy in 1986, it appeared that biliary lithotripsy in combination with oral dissolution therapy might be a valid alternative to cholecystectomy. In the experience of the largest lithotripsy centre in Munich, 80% of patients who have 1–3 non-calcified stones with a maximum diameter of 30 mm in a functioning gallbladder can be rendered stone free within 12 months. However, most other centres have yet to report their results.

In the first year of the lithotripsy programme, 16 patients with symptomatic cholelithiasis, who met the Munich criteria, have been treated at Southmead Hospital. Eleven patients had solitary stones, one had two stones, and four had three stones. Fragmentation was achieved in 11 of 15 patients in whom localisation was possible, after a mean of 1.3 sessions. Bile salt therapy (chenodeoxycholic acid 7 mg/kg; ursodeoxycholic acid 7 mg/kg) which was commenced 3 weeks prior to ESWL, has failed to produce complete dissolution in any patient after a mean of 5.6 months, although 3 failed to tolerate their medication. Three patients have required cholecystectomy for continuing symptoms and one for ascending cholangitis.

Although ESWL can usually fragment gallstones its ultimate success depends upon dissolution therapy. Experience has shown that this may need to be prolonged and is sometimes poorly tolerated.

* Abstract of Paper given at Symposium on Hepato-biliary Disease, Bristol Sep 89 and omitted from Report in June number.

### MANAGEMENT OF DISC BATTERY INGESTION IN CHILDREN

**N. M. El. Barghouty**
Yeovil District Hospital

Accidental ingestion of Disc Batteries by children is becoming an increasing problem facing medical practitioners since these batteries are used as energy sources for watches, hearing aids, calculators and cameras. They are present in most homes within the reach of children.

There are four main types of batteries, the most common and the most dangerous is the mercury cell battery as it contains a potentially toxic amount of mercury. Complications can arise if the battery is lodged in the gut or when it becomes corroded and releases its toxic content. These complications include oesophegeal perforation, tracheaoesophageal fistula and mercury poisoning. Management should include plain chest and abdominal x-ray on admission. If the battery is lodged in the oesophegus then immediate endoscopic removal is indicated. If the battery is in the stomach intact, conservative treatment is indicated but if the battery remains in the stomach for more than 24 hours or if there is radiological evidence of leakage, signs of peritoneal irritation or manifestation of mercury poisoning, then surgical or endoscopic removal is indicated and the blood mercury levels are measured which, if raised, the patient should be treated by a mercury cholating agent as dimercaphrol.

Fortunately only 9.9% of patients with disc battery ingestion develop symtoms, and following an accurate management protocol complications should be avoidable.

### SURGICAL SERVICES IN THE SOUTH WEST

**C. D. Collins**
Taunton

A study of the Performance Indicators collected by the Regional Information Unit indicated marked disparities in the distribution of surgical manpower and resources throughout the District General Hospitals in the South West Region. The proposal in the 'White Paper' that 100 extra consultants would be appointed in the surgical specialties was the stimulus to try to define where the need was greatest in the South West. It was clear that surgical beds and theatre sessions per consultant were greatest in Cheltenham and Torbay and fewest in Bristol and Weston. The discharges and deaths per consultant followed the same pattern. The disparity in workload was even more marked if the supporting junior staff were taken into account. Overall the staffing, both by consultants and by the total numbers of surgical staff in a DGH were considerably less per 100,000 population in all hospitals in the South West Region outside the BRI than in other Regions, notably South East Thames, Northern Region or, particularly, Scotland. If a 'norm' of one General Surgeon per 50,000 population is accepted with a half-share Registrar and SHO in support, then there is a requirement for 12 new General Surgical Consultant posts in the South West Region to meet this target and, in effect, to bring up the consultant per 100,000 population level towards that enjoyed by the South East Thames and the Northern Regions.

It was hoped to use arguments based on this data to encourage the early expansion of the consultant grade in general surgery and urology in the South West Region.

